# A Novel G Protein–Biased Agonist at the *δ* Opioid Receptor with Analgesic Efficacy in Models of Chronic Pain[Fn FN3]

**DOI:** 10.1124/jpet.119.258640

**Published:** 2020-02

**Authors:** Alexandra E. Conibear, Junaid Asghar, Rob Hill, Graeme Henderson, Eva Borbely, Valeria Tekus, Zsuzsanna Helyes, Josephine Palandri, Chris Bailey, Ingemar Starke, Bengt von Mentzer, David Kendall, Eamonn Kelly

**Affiliations:** School of Physiology, Pharmacology and Neuroscience, University of Bristol, Biomedical Sciences Building, University Walk, Bristol, United Kingdom (A.E.C., R.H., G.H., E.K.); Faculty of Pharmacy, Gomal University, Khyber Pakhtunkhwa, Pakistan (J.A.); PharmInVivo Ltd., Szentagothai Research Centre, Centre for Neuroscience and Department of Pharmacology and Pharmacotherapy, Medical School, University of Pécs, Pécs, Hungary (E.B., V.T., Z.H.); Department of Pharmacy and Pharmacology, University of Bath, Bath, United Kingdom (J.P., C.B.); and PharmNovo AB, Kungshamn, Sweden (I.S., B.v.M., D.K.)

## Abstract

Agonists at the *δ* opioid receptor are known to be potent antihyperalgesics in chronic pain models and effective in models of anxiety and depression. However, some *δ* opioid agonists have proconvulsant properties while tolerance to the therapeutic effects can develop. Previous evidence indicates that different agonists acting at the *δ* opioid receptor differentially engage signaling and regulatory pathways with significant effects on behavioral outcomes. As such, interest is now growing in the development of biased agonists as a potential means to target specific signaling pathways and potentially improve the therapeutic profile of *δ* opioid agonists. Here, we report on PN6047 (3-[[4-(dimethylcarbamoyl)phenyl]-[1-(thiazol-5-ylmethyl)-4-piperidylidene]methyl]benzamide), a novel G protein–biased and selective *δ* opioid agonist. In cell-based assays, PN6047 fully engages G protein signaling but is a partial agonist in both the arrestin recruitment and internalization assays. PN6047 is effective in rodent models of chronic pain but shows no detectable analgesic tolerance following prolonged treatment. In addition, PN6047 exhibited antidepressant-like activity in the forced swim test, and importantly, the drug had no effect on chemically induced seizures. PN6047 did not exhibit reward-like properties in the conditioned place preference test or induce respiratory depression. Thus, *δ* opioid ligands with limited arrestin signaling such as PN6047 may be therapeutically beneficial in the treatment of chronic pain states.

**SIGNIFICANCE STATEMENT:**

PN6047 (3-[[4-(dimethylcarbamoyl)phenyl]-[1-(thiazol-5-ylmethyl)-4-piperidylidene]methyl]benzamide) is a selective, G protein–biased *δ* opioid agonist with efficacy in preclinical models of chronic pain. No analgesic tolerance was observed after prolonged treatment, and PN6047 does not display proconvulsant activity or other opioid-mediated adverse effects. Our data suggest that *δ* opioid ligands with limited arrestin signaling will be beneficial in the treatment of chronic pain.

## Introduction

Treatment of chronic pain remains a significant medical challenge; in terms of analgesics, *µ* opioid ligands such as morphine are routinely, albeit inappropriately, prescribed at present. Although *µ* opioid receptor ligands are effective in treating acute, severe pain, they often lack efficacy in chronic pain states (Glajchen (2001), and their clinical utility in such states is limited due to the associated side effects, the onset of tolerance, and the abuse liability of this drug class.

Increasing evidence implicates the *δ* opioid receptor as an attractive therapeutic target for various forms of chronic pain and certain emotional disorders, including depression and anxiety (Pradhan et al., 2011). The use of pharmacological tools and genetic approaches has enhanced our understanding of *δ* receptor–mediated behaviors, with *δ* receptor agonists reported to be effective in preclinical models of chronic pain, including those for neuropathic pain, inflammatory pain, and cancer (Gavériaux-Ruff and Kieffer, 2011). In comparison with *µ* receptor agonists, *δ* receptor agonists are associated with a milder adverse effect profile with no respiratory depression (Gallantine and Meert, 2005), little or no gastrointestinal dysfunction (Gallantine and Meert, 2005; Feng et al., 2006), and the absence of physical dependence (Cowan et al., 1988). However, enthusiasm for the development of novel *δ* agonists has been lessened due to the potential for proconvulsive activity (Comer et al., 1993; Broom et al., 2002) as well as the development of analgesic tolerance (Pradhan et al., 2010) that has been reported for some *δ* agonists. Importantly, the proconvulsive liability and analgesic tolerance do not appear to be a common property of *δ* agonists; rather, these on-target adverse effects are thought to be ligand-specific (Gendron et al., 2016), suggesting that the development of a ligand that retains analgesic efficacy but lacks these adverse effects is a plausible approach. As such, interest is now growing in the development of positive allosteric modulators (Burford et al., 2015) or biased agonists (Audet et al., 2012; Charfi et al., 2015) as a potential means to improve the therapeutic profile of *δ* opioid receptor agonists.

Biased agonism is now a well documented phenomenon whereby different ligands acting at the same receptor can stabilize distinct receptor conformations such that only a subset of the possible signaling pathways are activated relative to the signaling pathways activated by a reference ligand, normally a well known and studied full agonist ligand (Kelly, 2013; Kenakin and Christopoulos, 2013). Biased agonists will thus generate distinct signaling outputs and potentially different in vivo effects. Certain *δ* opioid agonists have already been developed that reportedly display an improved therapeutic profile. For example, JNJ-20788560 has been suggested not to induce analgesic tolerance (Codd et al., 2009), and ADL5859 does not exhibit proconvulsive activity even at doses over 300-fold greater than that required for its antihyperalgesic action (Le Bourdonnec et al., 2008). However, the potential signaling mechanisms underlying these differential effects have yet to be comprehensively assessed. For the *δ* receptor, it has been postulated that G protein–biased agonists may offer an approach to develop ligands that are effective in chronic pain states and emotional disorders but with a reduced adverse effect profile (Pradhan et al., 2011; Dripps et al., 2018). With respect to arrestin-mediated signaling from the *δ* opioid receptor, there is mounting evidence to implicate arrestin-mediated internalization with the development of analgesic tolerance. Several studies to date have demonstrated that low-internalizing agonists, including ARM390 and KNT-127, have a reduced propensity to induce desensitization and acute analgesic tolerance (Nozaki et al., 2014; Pradhan et al., 2016). In contrast, the high-internalizing agonist SNC80 desensitizes the receptor, resulting in analgesic tolerance as well as tolerance to other *δ* opioid–mediated behaviors (Pradhan et al., 2010). The signaling pathway(s) that underlies the proconvulsive activity of certain *δ* opioid agonists is still poorly understood. There are data to suggest that low-internalizing agonists have a decreased tendency to induce convulsions (Pradhan et al., 2011), yet SNC80-induced convulsions were unaffected in arrestin-3 knockout mice and actually potentiated in arrestin-2 knockout mice (Dripps et al., 2018). However, convergent data do imply that different signaling pathways contribute to the desired therapeutic effect over the adverse effects, suggesting that the *δ* opioid receptor is an attractive target for developing biased ligands or even ligands that only activate a subset of the receptor’s signaling repertoire.

In this study, we report on PN6047 (3-[[4-(dimethylcarbamoyl)phenyl]-[1-(thiazol-5-ylmethyl)-4-piperidylidene]methyl]benzamide), a novel and selective *δ* opioid receptor agonist structurally derived from SNC80. The current studies were conducted both to evaluate the in vitro signaling profile and to determine any potential bias as well as the behavioral effects of PN6047 in vivo. We found that, relative to SNC80, PN6047 is a G protein–biased *δ* agonist with potent antihyperalgesic efficacy in different models of chronic pain, as well as exhibiting a diminished adverse effect profile.

## Materials and Methods

### Reagents

**Fig. 1.**
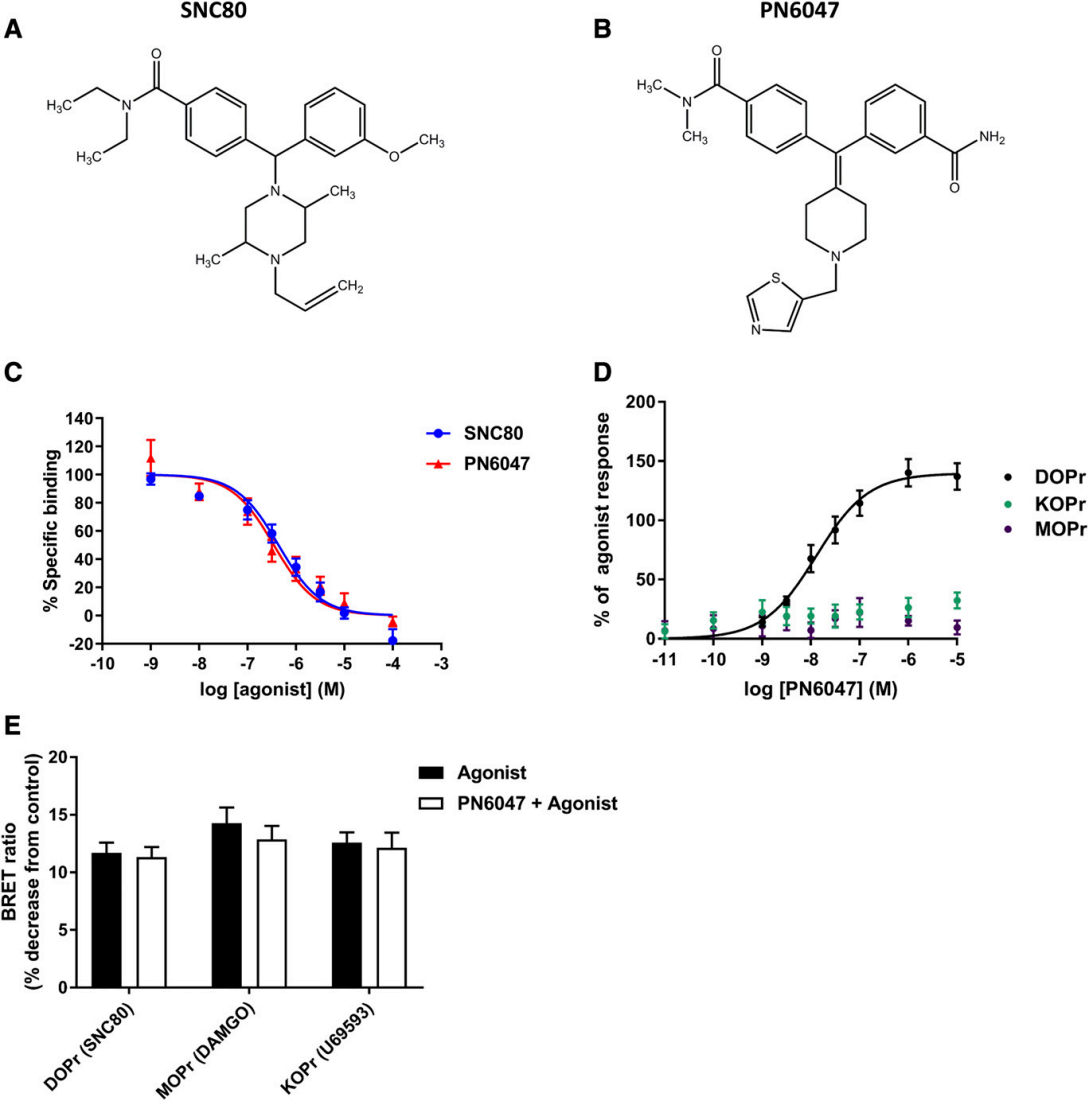
PN6047 is a selective *δ* opioid receptor agonist. Structure of SNC80 (A) and PN6047 (B). (C) Competitive inhibition of [^3^H]-diprenorphine (2 nM) binding in HEK-DOR membranes by SNC80 or PN6047. Nonspecific binding was determined using naltrindole (30 *µ*M). Data represent the mean ± S.E.M. (*n* = 4). (D and E) Opioid‐induced G*α*_i_ protein activation measured using BRET technology in HEK293 cells expressing recombinant opioid receptors together with G*α*_i_-Rluc, G*γ*-GFP, and G*β*. Concentration response to PN6047 in cells expressing either the *δ*, *µ*, or *κ* opioid receptors (D). Cells were also stimulated with 10 *µ*M prototypical agonist for the respective receptor (*δ*, SNC80; *µ*, DAMGO; *κ*, U69593) to elicit a near-maximal response (E). The averaged response to these prototypical agonists was taken as 100% for each receptor to normalize the response to PN6047 (D). In the same experiments, cells were preincubated with PN6047 (10 *µ*M) for 2 minutes prior to stimulation with 10 *µ*M prototypical agonist for the respective receptor to assess any antagonistic activity (E). Data represent the mean ± S.E.M. (*n* = 4–6). Data in (E) were analyzed by Student’s *t* test for comparison between the response elicited by each of the agonists in the absence and presence of PN6047. KOPr, *κ* opioid receptor; MOPr, *µ* opioid receptor.

cDNA encoding the wild-type human hemagglutinin (HA)–*δ* opioid receptor (DOPr) and human G*β*1 were obtained from the Missouri University of Science and Technology (http://www.cdna.org). The G protein bioluminescence resonance energy transfer (BRET) constructs were a gift from Michel Bouvier (University of Montreal, Montreal, Canada), and the *δ* receptor–renilla luciferase and arrestin‐3 and -2 Green Fluorescent Protein (GFP) constructs were a gift from Dr. Tomasso Costa (Istituto Superiore di Sanità, Rome, Italy). PN6047, shown in Fig. 1B, was synthesized by PharmNovo AB. The synthesis of PN6047 was performed according to the patent WO2016/099393, with US patent number US 10,118,921 B2. SNC80, [D-Ala^2^, D-Leu^5^]-enkephalin (DADLE), and ARM390 were obtained from Tocris. Methadone and heroin were from Macfarlan Smith (Edinburgh, UK). Cmpd101 was from Hello Bio. [^3^H]-Diprenorphine was from PerkinElmer. Coelenterazine 400a was purchased from Biotium. The phosphorylated ERK (pERK) antibody was from Cell Signaling Technology, and antitubulin was from Sigma-Aldrich.

### Animals

All animal care and experimental procedures undertaken in the United Kingdom were performed in accordance with the UK Animals (Scientific Procedures) Act 1986 and the European Communities Council Directive (2010/63/EU). For experiments conducted by PharmInVivo Ltd., University of Pécs (Pécs, Hungary), all experimental procedures were carried out according to the 1998/XXVIII Act of the Hungarian Parliament on Animal Protection and Consideration Decree of Scientific Procedures of Animal Experiments (243/1988). The experiments were approved by the Ethics Committee on Animal Research of Pécs University according to the Ethical Code of Animal Experiments (Ethical licence numbers are BA 02/2000-9/2011; BA 02/2000-25/2011; BA 02/2000-2/2012). Details regarding species and strain used are specified within each experimental procedure.

### In Vitro Studies

#### Cell Culture.

Human embryonic kidney 293 (HEK293) cells were maintained in Dulbecco’s modified Eagle’s medium (DMEM) supplemented with 10% FBS and 100 U/ml penicillin-streptomycin at 37°C in a humidified atmosphere of 95% air, 5% CO_2_. For transient transfection, HEK293 cells were seeded onto 10-cm dishes and grown to 80% confluency before transfection using Lipofectamine 2000 (Invitrogen). For the generation of a HEK-DOPr stable cell line, clonal cells were obtained by limited dilution, and stable clones were selected in the presence of 600 *μ*g/ml geneticin and maintained in the presence of 400 *μ*g/ml geneticin. Single clones were isolated after 2 to 3 weeks of culture and screened for expression by surface receptor ELISA and radioligand binding assays. CHO-K1-hDOR and U20S-OPRD1 cells were purchased from DiscoverX, and cells were maintained in DMEM/F12 containing 2 mM l-glutamine, 10% FBS, and 100 U/ml penicillin-streptomycin.

#### Radioligand Binding Assays.

Cells were grown in 75-cm^2^ flasks until approximately 90% confluent. The cells were washed twice with ice-cold PBS before being dislodged with ice-cold hypotonic lifting buffer [10 mM HEPES, 0.9% (w/v) NaCl, 0.2% (w/v) EDTA, pH 7.4], and pelleted by centrifugation at 377*g* for 10 minutes at 4°C. Membranes were prepared as previously described (McPherson et al., 2010). For saturation binding experiments, 10 *μ*g of membrane protein was incubated with increasing concentrations of [^3^H]-diprenorphine (0.06–30 nM). For competition binding experiments, 2 nM [^3^H]-diprenorphine was incubated in the presence of increasing concentration of agonist (1 nM to 100 *µ*M). Nonspecific binding was determined by carrying out parallel determinations in the presence of excess unlabeled naltrindole (30 *µ*M). All binding reactions were prepared in 500 *μ*l volumes of assay buffer [Hanks’ buffered saline solution also containing 20 mM (4-(2-hydroxyethyl)-1-piperazineethanesulfonic acid) (HEPES) at pH 7.4] and performed in duplicate. The binding reaction was allowed to proceed for 90 minutes at room temperature with gentle agitation. Reactions were terminated by the addition of 3 ml of ice-cold wash buffer (20 mM HEPES, pH 7.4) and rapid filtration under vacuum through glass fiber (GF/B) filters using a Brandel cell harvester. The amount of bound [^3^H]-diprenorphine to membranes on individual filters was quantified by liquid scintillation counting.

#### BRET Assays.

To determine the relative ability of the agonists to activate G*α*_i_ G proteins, a BRET^2^-based assay that monitors the separation of G*α*_i1_ and G*γ*2 was used. In this configuration, a decrease in the BRET ratio is used as a measure of G protein activation. HEK293 cells were transiently transfected with HA-DOPr, G*α*i1–Renilla Luciferase II (RlucII), GFP_10_-G*γ*2, and G*β*1. To determine the extent of agonist-induced arrestin recruitment, cells were cotransfected with human DOPr-Rluc and either arrestin-2 or 3-GFP. Immediately prior to each assay, cells were resuspended in phenol red free DMEM and then transferred to a 96-well plate at 90 *μ*l per well. Measurements of BRET were made at 37°C. Coelenterazine 400a, at a final concentration of 5 *μ*M, was injected 5 seconds prior to reading the cell plate. BRET measurements were obtained on a FLUOstar Omega plate reader (BMG LABTECH, Ortenberg, Germany) using the following filter set: acceptor, 515 ± 30 nm filter; and donor, 410 ± 80 nm filter. BRET signals were determined as the ratio of the light emitted by acceptors (GFP_10_) over donor (RlucII). For G*α*_i_ activation, BRET measurements were taken 2 minutes after agonist application and 10 minutes after agonist application for arrestin recruitment.

#### ERK Assay.

Western blot analysis of pERK was performed as described previously (Cooke et al., 2015). For quantification, pERK levels were normalized against corresponding tubulin levels determined in the same experiment, which served as a loading control. Densitometry of bands was undertaken using ImageJ (National Institutes of Health).

#### cAMP.

Levels of cAMP accumulation were determined using the HitHunter cAMP XS+ Assay kit (DiscoverX). CHO-K1-hDOR cells were incubated with agonists for 48 hours. On the day of assay, agonist-treated cells were given a 30-minute washout period. The cells were re-exposed to the agonists and then subsequently challenged with forskolin (1 *µ*M) in the presence of rolipram (1 *µ*M), and the plates were incubated for 30 minutes at room temperature. The chemiluminescent signal was detected using a TopCount NXT Plate reader (PerkinElmer). The data were expressed as a percentage of the forskolin response.

#### Internalization.

Internalization was assessed using the PathHunter GPCR Internalization Assay kit (DiscoverX) according to the manufacturer’s instructions. U2OS-OPRD1 cells were exposed to agonists for 3 hours at 37°C. Chemiluminescence, indicated as relative luminescence units, was measured on a TopCount NXT Plate reader (PerkinElmer).

### In Vivo Studies

#### Mechanical Hyperalgesia and Acute Inflammatory Nociception Studies.

These studies were conducted by PharmInVivo Ltd., University of Pécs.

##### Sciatic nerve ligation.

Neuropathic pain was induced in either male NMRI mice (30–45 g) or male Wistar rats (237–310 g) using the sciatic nerve ligation (SNL) procedure as previously described (Seltzer et al., 1990; Malmberg and Basbaum, 1998). Initially, animals were conditioned to the experimental apparatus (Fig. 3A, day −4), and then three baseline measurements were taken on three consecutive days (Fig. 3A, days −3, −2, −1). Animals were then anesthetized with a combination of ketamine (100 mg/kg, i.p.) and xylazine (10 mg/kg, i.p.). Traumatic mononeuropathy on the right hindlimb was induced by tight ligation of 1/3 of the sciatic nerve. The mechanonociceptive thresholds of the plantar surface of the paw were measured with a dynamic plantar aesthesiometer. The paw withdrawal threshold was obtained in grams (maximal value 10 g, ramp time 4 seconds). Hyperalgesia (decrease of the withdrawal thresholds) was expressed as percentage by comparing the data of each individual animal to the averaged baseline threshold established prior to injury on days −3, −2, and −1 (Fig. 3A). Animals that did not develop the minimum of 20% hyperalgesia in response to SNL were excluded from the studies.

##### Mono-iodoacetate–induced osteoarthritic pain model.

Male NMRI mice (30–45 g) were anesthetized with a combination of ketamine (100 mg/kg, i.p.) and xylazine (10 mg/kg, i.p.). Mono-iodoacetate (MIA; 25 mg/ml in 20 *µ*l) was injected in the right knee joint and saline (20 *µ*l) in the left knee joint as a control, as described previously (van Osch et al., 1994). On the seventh day after MIA injection, mice were treated orally with PN6047 (10 mg/kg) or vehicle [hydroxypropyl methylcellulose (HPMC)], and paw withdrawal thresholds were measured 60 minutes later. The mechanonociceptive threshold of the plantar surface of the hind paws was measured with dynamic plantar aesthesiometry. Hyperalgesia was expressed as a percentage of initial control values as determined from the baseline measurements taken prior to MIA injection as described earlier. Mice with a minimum of 20% pretreatment hyperalgesia were not included in the study.

##### Carrageenan-induced acute inflammation and inflammatory pain.

Sixty minutes after oral administration of 1, 3, or 10 mg/kg PN6047 or vehicle (HPMC), carrageenan (3%, 50 *μ*l) was injected into the plantar surface of one hind paw to induce subacute inflammatory hyperalgesia. The mechanonociceptive threshold was determined with dynamic plantar aesthesiometry as described earlier, and paw volume was determined with plethysmometry. Measurements were taken at 2, 3, 4, and 24 hours after carrageenan the treatment.

#### Behavioral Studies and Adverse Effect Assessment.

##### Forced swim test.

Male NMRI mice (30–45 g) were administered PN6047 [10 mg/kg, by mouth (PO)] or vehicle (HPMC, PO). One hour post drug administration, mice were placed in a water-filled cylinder, and immobility time was recorded manually during the last 4 minutes of the 6-minute experimental period.

##### Intravenous pentylenetetrazol seizure threshold.

This study was conducted by Transpharmation Ltd. CD-1 male mice (28–34 g) were pretreated with vehicle (HPMC, PO) or PN6047 (80 mg/kg, PO) for 30 minutes. Mice were then lightly restrained and injected intravenously with pentylenetetrazol (8 mg/ml in 0.9% saline) via a butterfly cannula placed in a superficial tail vein. Pentylenetetrazol (PTZ) was infused at a constant rate (0.5 ml/min). Latencies to onset of first myoclonus, forelimb tonus, and/or hindlimb tonic extension were recorded. Infusions were stopped at the appearance of hindlimb tonic extension up to a cutoff of 120 seconds. For animals reaching the cutoff, the dose of PTZ in milligrams per kilogram infused over the 120 seconds was calculated as the threshold dose. The threshold doses of PTZ (milligrams per kilogram) required to produce the different seizure types were calculated according to the following formula:
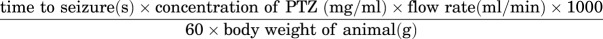


##### Conditioned place preference and locomotor activity.

This study was conducted by RenaSci (Nottingham, UK). Place preference conditioning was conducted in a conditioned place preference (CPP) chamber with an auto monitoring system (EthoVision XT version 8.0; Tracksys, Nottingham, UK). The apparatus consisted of a two-compartment box (40 × 40 cm each) with different walls and floors, covered with sound-attenuating material (Wright et al., 2019). Experiments were performed in dim white light (approximately 15 lux).

Male Wistar rats (6 to 7 weeks old) were habituated to laboratory conditions for 4 days prior to experimentation. On days 5 and 6, rats were habituated to the entire chamber for 15 minutes (one session/day). On days 7–10, rats were conditioned (40 minutes) to one of the two compartments and received saline (1 ml/kg, s.c.), heroin (1 mg/kg, s.c.), PN6047 (3 mg/kg, i.p.), PN6047 (9 mg/kg, i.p.), or HPMC vehicle (1.5 ml/kg, i.p.) On the following day, those that received heroin or PN6047 received saline or HPMC vehicle and were restricted to the opposite side of the CPP apparatus. This was repeated over 4 days, so rats all received two injections of drug and two injections of vehicle during the conditioning sessions. The order of injections within each group was selected randomly. Twenty-four hours after the last conditioning day, the guillotine doors were removed, and animals were allowed to roam freely in the CPP apparatus for 15 minutes. The time spent in each chamber and the total distance moved were recorded and analyzed using the EthoVision software.

For CPP, data throughout are presented after multiplying by a correction factor. The correction factor was calculated by dividing duration of test (900 seconds) by total time spent in the two large compartments. This factor is used to proportionally divide the time spent in the neutral central compartment between the two conditioning compartments. The conditioned place preference scores are presented as time spent in the drug-paired side for 450 seconds (half of the post-test time) to give a preference score that represents an increase in time spent in the drug-paired side.

##### Respiration depression and tail-flick latency.

For acute antinociception and respiratory depression experiments, male CD-1 mice (28–32 g) were used. Respiration, measured as minute volume, was monitored using whole-body plethysmography as described previously (Hill et al., 2018). Antinociception was measured as an increase in tail flick latency using the warm-water (52.5°C) tail-flick assay with a cutoff of 15 seconds. Mice were habituated to plethysmograph chambers as well as general handling for 30 minutes on the day prior to experimentation. On the day of the experiment, tail-flick latency was measured in mice before and after measurement of a respiratory baseline for 20 minutes. Following the second tail-flick measurement, vehicle (HPMC), methadone (10 mg/kg), or PN6047 (20 mg/kg) was administered orally. Respiration was monitored for 10 minutes after drug administration, and mice were then removed from the chambers for the measurement of tail-flick latency. Mice were returned to their cages for 10 minutes before another tail-flick latency was measured, and mice were returned to the chamber for measurement of respiration for a further 10 minutes. This process of measurements was repeated until 70 minutes post administration.

### Data Analysis

All values are expressed as the mean ± S.E.M. Concentration-response data were analyzed using nonlinear curve fitting (GraphPad Prism Software; GraphPad Software, San Diego, CA) to obtain EC_50_ and E_max_ values for G protein activation, cAMP accumulation, and arrestin recruitment, or IC_50_, pK_i_, and B_max_ values from radioligand binding assays. Agonist bias was quantified using the operational model as previously reported (Kenakin et al., 2012). Concentration-response curves were fitted to the Black-Leff operational model to determine transduction coefficients (*τ*/K_A_). Ligand bias factors (Δlog[*τ*/K_A_]) are expressed after normalization against the prototypical *δ* opioid agonist SNC80, used as the reference ligand. Bias factors were then expressed as the ΔΔlog[*τ*/K_A_] value between two different signaling pathways. For statistical analysis, the ΔΔlog[*τ*/K_A_] values were transformed to the corresponding antilog value.

Statistical analyses used in each experiment are detailed in respective figure legends. In brief, comparisons of agonist efficacy, potency, and bias factors were quantified using one-way ANOVA followed by Bonferroni’s post hoc test. Student’s *t* test was used to assess potential antagonistic activity of PN6047 at the *µ* and *κ* opioid receptors. The SNL data, carrageenan-induced acute inflammation and inflammatory pain, and proconvulsive liability data sets were analyzed by two-way ANOVA followed by Bonferroni’s modified *t* test for comparisons between either pre- and post-treated values or vehicle- and drug-treated groups, as indicated. The data from the MIA model were analyzed by one-way ANOVA followed by Bonferroni’s modified *t* test to compare the pre- and post-treatment values. Data from the forced swim test and CPP experiments were analyzed with Student’s *t* test for paired comparison between the pre- and post-treated values or postconditioning and habituation scores, respectively. The respiratory depression and tail-flick assays were assessed by either one-way or two-way ANOVA as indicated, followed by Bonferroni’s post hoc test.

## Results

### In Vitro Studies

To determine the affinity of PN6047 (Fig. 1C), competition binding studies were performed with PN6047 and SNC80 using the nonselective opioid receptor ligand [^3^H]-diprenorphine as the label in the presence of a physiologic concentration of sodium ions. Binding was assessed in membranes prepared from HEK293 cells stably expressing an HA-tagged *δ* opioid receptor (Fig. 1C). Receptor expression levels were determined by saturation binding assays with [^3^H]-diprenorphine, and the B_max_ of binding was found to be 1.91 ± 0.29 pmol/mg protein. For competition binding studies, SNC80 and PN6047 were fit by nonlinear regression to a single site competition model and were found to have a similar affinity for the *δ* opioid receptor (SNC80 pK_i_ 7.06 ± 0.17; PN6047 pK_i_ 7.25 ± 0.10). To assess if PN6047 has efficacy at other members of the opioid receptor family, we used a G protein BRET assay. The opioid receptor family members are primarily coupled to G*α*_i_ that mediate, among other things, inhibition of adenylyl cyclase. HEK293 cells were transiently transfected with the *δ*, *µ*, or *κ* opioid receptors together with G*α*_i_-Rluc, G*β*, and G*γ*-GFP. PN6047 elicited a concentration-dependent activation of G*α*_i_ G proteins in cells expressing the human *δ* opioid receptor (Fig. 1D; EC_50_ 13.5 ± 2.9 nM). In cells expressing either the rat *µ* opioid receptor or human *κ* opioid receptor, PN6047 had no effect on G*α*_i_ activation. In the same experiment, cells were also preincubated with or without PN6047 (10 *µ*M; 2 minutes) prior to stimulation with 10 *µ*M prototypical agonist for each of the opioid receptors (*δ*, SNC80; *µ*, DAMGO ([D-Ala^2^, *N*-MePhe^4^, Gly-ol]-enkephalin); *κ*, U69593) to determine a near-maximal effect for each of the receptor constructs and investigate any potential antagonist activity of PN6047 at either the *µ* opioid receptor or *κ* opioid receptor. PN6047 had no effect on the ability of either DAMGO or U69593 to elicit a response (Fig. 1E), suggesting that PN6047 does not display antagonist activity at the other opioid receptors. Initially, experiments were undertaken using a human *µ* opioid receptor construct; however, we were unable to achieve a strong signal with this construct even in response to DAMGO (10 *µ*M), making the effect window too small for accurate analysis.

**Fig. 2.**
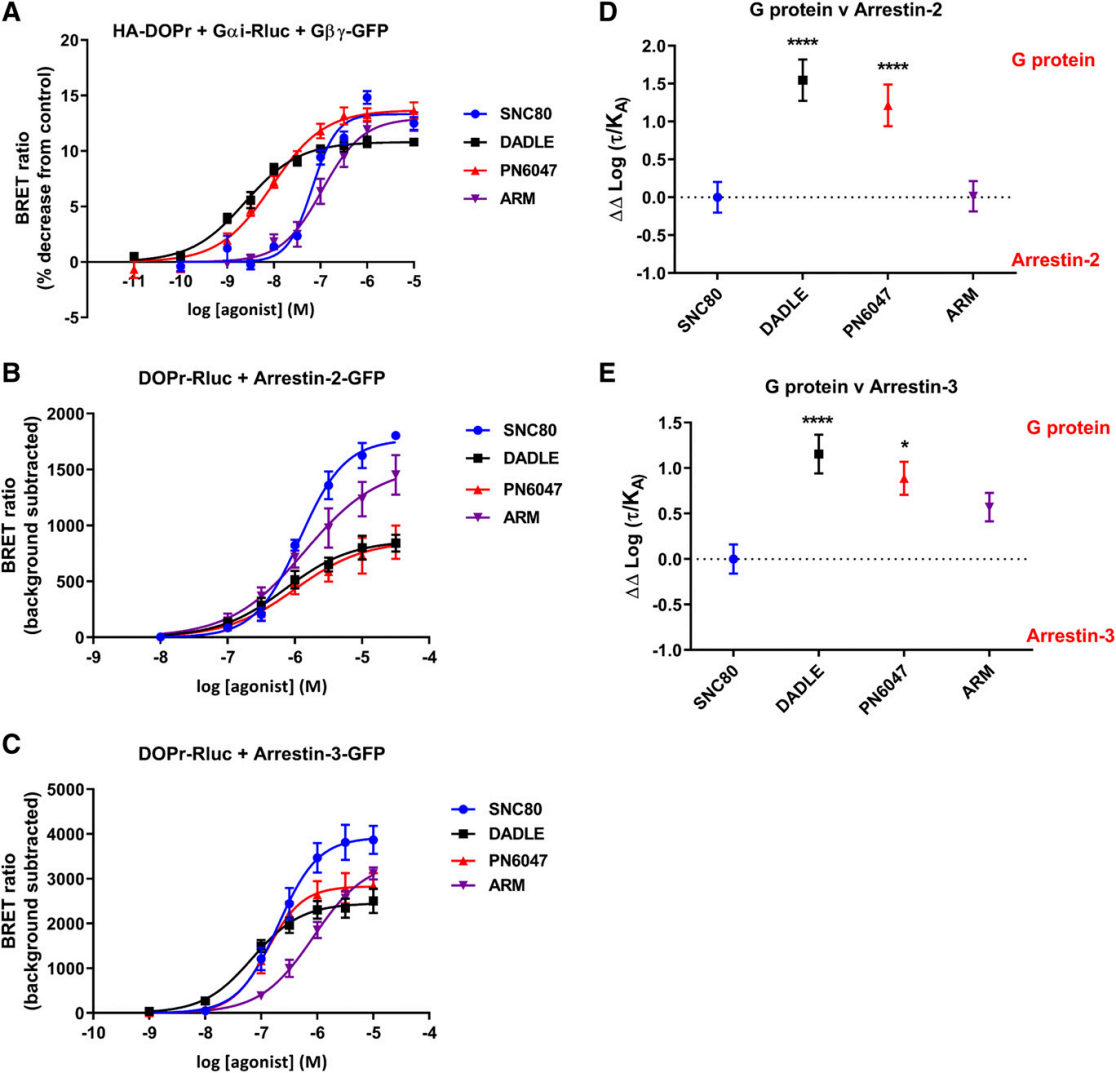
PN6047 is a G protein–biased agonist. (A) Activation of G*α*i G proteins. (B and C) Recruitment of arrestin-2 and arrestin-3, respectively, measured as a change in BRET ratio. HEK293 cells were transiently transfected with HA-tagged DOPr, G*α*_i_-RlucII, G*γ*-GFP, and G*β* for the G protein BRET studies or DOPr-Rluc and arrestin-2/3-GFP to measure arrestin recruitment. Cells were stimulated with agonist for either 2 minutes [(A); G protein subunit dissociation] or 10 minutes [(B and C); arrestin-2 or -3 recruitment] before the addition of 5 *μ*M coelenterazine 400a, and changes in BRET ratio were measured. Data represent the mean ± S.E.M. (*n* = 5–7). (D and E) Bias factors for all agonists between G protein activation and arrestin recruitment relative to SNC80. *P < 0.05; ****P < 0.0001, as determined by one-way ANOVA followed by Bonferroni’s post hoc test.

To characterize the in vitro signaling profile of PN6047 and assess any potential bias, we investigated the ability of PN6047 to activate G*α*_i_ or recruit either arrestin-2 or arrestin-3 using BRET technology. The signaling profile of PN6047 was compared with that of three widely studied *δ* opioid receptor agonists (SNC80, DADLE, and ARM390). Concentration-response curves for ligand-induced changes in BRET ratios were analyzed to determine the potency (EC_50_) and the maximum response (E_max_) of each ligand. The data for these signaling outputs are summarized in Table 1. As shown in Fig. 2A, SNC80, PN6047, and ARM390 were observed to be full agonists in the G protein BRET assay; the maximum response to DADLE was considered statistically different from that of SNC80, suggesting that in this assay, DADLE is a partial agonist. The potency order of the four agonists was DADLE = PN6047 > SNC80 > ARM390.

**TABLE 1:** Summary of binding affinities, potencies, and maximal effects for DADLE, SNC80, PN6047, and ARM390 in the G*α*_i_ coupling and arrestin‐2 and -3 recruitment assays Shown are the mean ± S.E.M.

	[^3^H]-Diprenorphine		G Protein				Arrestin-2				Arrestin-3			
	pKi	S.E.M.	LogEC_50_	S.E.M.	E_max_	S.E.M.	LogEC_50_	S.E.M.	E_max_	S.E.M.	LogEC_50_	S.E.M.	E_max_	S.E.M.
DADLE	N.D.	N.D.	−8.61	0.12	10.84*	0.3	−6.08	0.17	880.233*	65.6	−7.16	0.05	2467.2*	247.9
SNC80	7.06	0.17	−7.13	0.04	13.4	0.6	−5.94	0.01	1675.6	105.2	−6.67	0.06	3924.6	327.7
PN6047	7.25	0.1	−8.05	0.10	13.8	0.8	−5.98	0.08	868.5*	165.7	−6.84	0.10	2847*	285.2
ARM390	N.D.	N.D.	−6.96	0.12	13.0	0.9	−5.92	0.21	1502.9	223.4	−6.08	0.07	3371.4	133.7

N.D., not determined.

* *P* < 0.05, significantly different from the SNC80 maximum value (one-way ANOVA with Bonferroni’s post hoc test).

We then examined the recruitment of either arrestin-2 or arrestin-3 to the *δ* opioid receptor using a BRET assay. All agonists stimulated recruitment of both arrestins in a concentration-dependent manner. Analysis of the maximum response of each agonist to recruit either arrestin-2 or arrestin-3 (Fig. 2, B and C; Table 1) revealed that both PN6047 and DADLE were partial agonists for arrestin-2 and arrestin-3 recruitment, with significantly lower efficacy than SNC80. In comparison with the G protein assay, the potencies for the four agonists to recruit arrestin-2 were not substantially different, with all agonists having an EC_50_ in the low-micromolar range. For arrestin-3 recruitment, the rank order of potencies was DADLE = PN6047 = SNC80 > ARM390.

To quantify biased agonism at these signaling pathways, bias factors were calculated as described in *Materials and Methods*. Quantification of bias between each pathway was performed using SNC80 as the reference ligand. Both PN6047 and DADLE showed significant bias toward G protein activation over recruitment of arrestin-2 or arrestin-3, compared with SNC80 (Fig. 2, D and E). ARM390 was not significantly biased for either G protein activation or arrestin recruitment. Direct comparison of the concentration-response curves for SNC80 and PN6047 (Supplemental Fig. 1) clearly highlights the G protein–biased profile of PN6047 relative to SNC80, with the position of the PN6047 curve being to the left of the SNC80 curve for G protein activation but to the right of SNC80 for arrestin recruitment.

To investigate signaling and regulation further downstream of receptor activation, we determined the ability of PN6047 and SNC80 to activate ERK and induce internalization, and we also assessed the contribution of GRK2/3 to arrestin recruitment. Both ligands at 10 *µ*M activated ERK in a time-dependent manner to a similar extent (Supplemental Fig. 2, A and B). For the *δ* opioid receptor, activation of ERK is reportedly downstream of G protein activation (Gendron et al., 2016). Trafficking of the *δ* opioid receptor is known to be ligand-dependent and purportedly important for differential behavioral effects in vivo (Pradhan et al., 2009, 2010). While both SNC80 and PN6047 induced internalization of the *δ* opioid receptor, the potency and efficacy of SNC80 was greater than that of PN6047 (Supplemental Fig. 2C). In BRET-based arrestin recruitment assays, preincubation with compound 101, a GRK2/3 selective inhibitor, attenuated both arrestin-2 and arrestin-3 recruitment. The extent of inhibition of arrestin recruitment was similar for SNC80 and PN6047 in both arrestin-2 and arrestin-3 recruitment assays (Supplemental Fig. 2, D and E).

### Antinociception Studies

#### SNL Model.

**Fig. 3.**
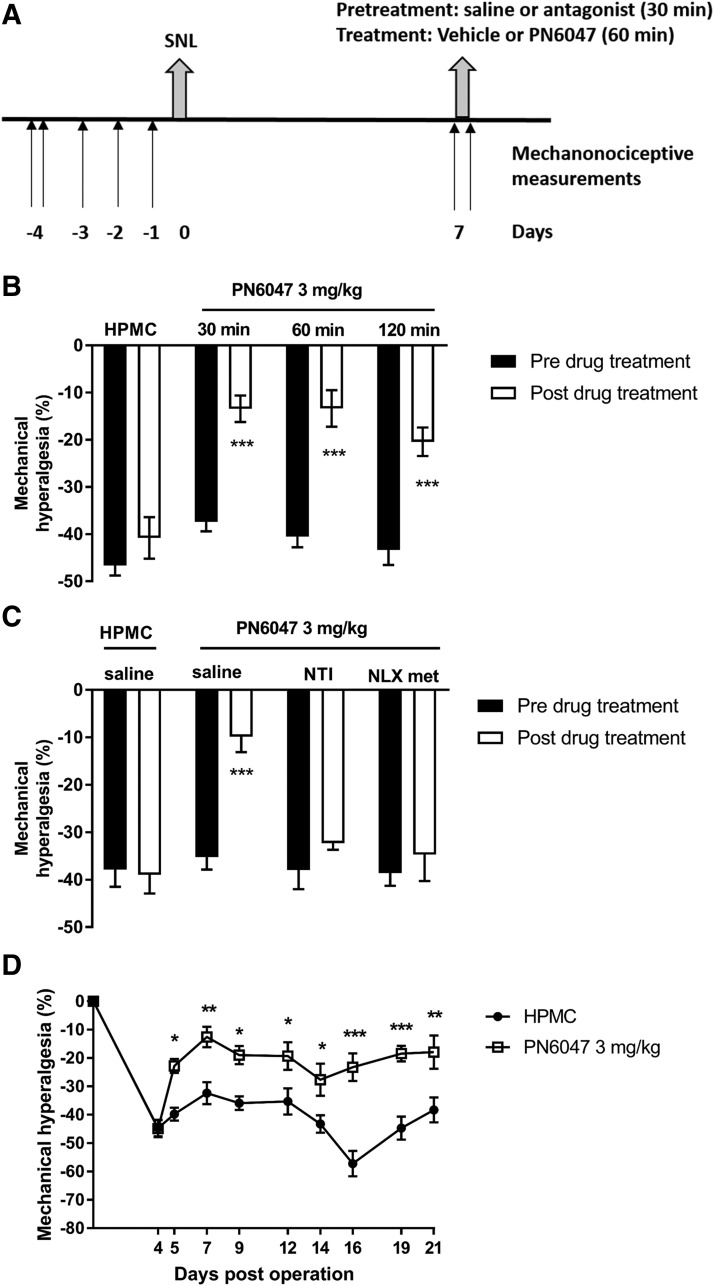
PN6047 is an effective antihyperalgesic in the SNL model of neuropathic pain. (A) Schematic of the SNL experimental procedure as detailed in *Materials and Methods*. Arrows indicate the days on which mechanonociceptive measurements were taken, with negative values representing presurgery days when a conditioning (day −4) and three baseline measurements were acquired over three consecutive days (−3, −2, −1). (B) Time course of PN6047 antihyperalgesic effect in the mouse SNL model. Mechanonociceptive thresholds were measured 7 days post surgery; vehicle (HPMC, PO) or PN6047 (3 mg/kg, PO) was administered; and mechanonociceptive thresholds were assessed at 30, 60, and 120 minutes post administration (*n* = 5–8 mice/group). (C) Antagonism of PN6041-induced hyperalgesia with opioid antagonists in the mouse SNL model. Mechanonociceptive thresholds were determined prior to pretreatment with either saline, naltrindole (NTI; 3 mg/kg, i.p.), or naloxone methiodide (NLX met; 8 mg/kg, i.p.) for 30 minutes. Animals were then administered vehicle (HPMC, PO) or PN6047 (3 mg/kg; PO) and mechanonociceptive thresholds were determined 60 minutes later (*n* = 7 to 8 mice/group). (D) Repeated dosing of PN6047 retains antihyperalgesic activity in the mouse SNL model. Animals were dosed every 24 hours with vehicle (HPMC, PO) or PN6047 (3 mg/kg, PO), and mechanonociceptive thresholds were determined 60 minutes following drug administration on days 5, 7, 9, 12, 14, 16, 19, and 21 post surgery. Results are expressed as the mean ± S.E.M. of the mechanonociceptive threshold changes (*n* = 8 to 9 mice/group). Data in (B) and (D) were analyzed by two-way repeated-measures ANOVA followed by Bonferroni’s modified *t* test to compare either the pre- and post-treatment values (B) or the drug effect to the vehicle at respective time points (D). Data in (C) were analyzed by two-way ANOVA followed by Bonferroni’s modified *t* test to compare the pre- and post-treatment values (*P < 0.05; **P < 0.01; ***P < 0.001).

In the murine SNL model of neuropathic pain (Fig. 3A), PN6047 at 3 mg/kg (PO) significantly reversed the mechanical hyperalgesia for at least 2 hours (Fig. 3B). A schematic of the experimental procedure is detailed in Fig. 3A. No changes in the mechanonociceptive thresholds were detected on the contralateral paw across the different experiments (data not shown). The dose of PN6047 was selected as one producing a robust antihyperalgesic effect as determined in the rat SNL model (Supplemental Fig. 3). The antihyperalgesic action of PN6047 was retained at 30 (*P* < 0.001), 60 (*P* < 0.001), and 120 minutes (*P* < 0.001) post PN6047 administration. The antihyperalgesic effect of PN6047 did not differ significantly across the time points investigated, demonstrating that the antihyperalgesic action is retained for at least 2 hours. Mechanonociceptive thresholds were not altered by vehicle (HPMC) 60 minutes post administration (Fig. 3B).

The antihyperalgesic action of PN6047 in the mouse SNL model was reversed by the selective *δ* opioid antagonist naltrindole hydrochloride as well as by the peripherally restricted, nonselective opioid antagonist naloxone methiodide (Fig. 3C). Treatment with PN6047 (3 mg/kg, PO, 60 minutes) induced robust antihyperalgesia (*P* < 0.001). Pretreatment for 30 minutes with either naloxone methiodide (8 mg/kg, i.p.) or naltrindole hydrochloride (3 mg/kg, i.p.) significantly inhibited the antihyperalgesic effect of PN6047 (Fig. 3C). This indicates a *δ* receptor–mediated effect with a peripheral *δ* receptor component for the antihyperalgesic effect of PN6047 in the SNL model.

#### Tolerance Evaluation.

The potential for the development of tolerance to the analgesic effectiveness of PN6047 was studied using the SNL model in mice. PN6047 retained antihyperalgesic efficacy following repeated dosing in the SNL model (Fig. 3D). PN6047 (3 mg/kg, PO) was administered once daily for 16 days to mice subjected to SNL 5 days previously. Mechanonociceptive measurements were assessed 60 minutes following drug administration on days 5, 7, 9, 12, 14, 16, 19, and 21 post surgery. Treatment with PN6047 resulted in a significant inhibition of hyperalgesia on the first day of treatment (−39.7% ± 2.2% vs. −22.7% ± 2.5% hyperalgesia, vehicle vs. PN6047, respectively; *P* < 0.05). The antihyperalgesic effect of PN6047 was maintained during the 16-day dosing period (−38.2% ± 4.4% vs. −17.8% ± 5.9% hyperalgesia, vehicle vs. PN6047, respectively, day 21; *P* < 0.01). Thus, tolerance to the antihyperalgesic effect of PN6047 does not appear to develop in this model, with the compound continuing to reverse the SNL-induced hyperalgesia after 16 days of dosing.

Interestingly, in vitro measures of desensitization demonstrated that PN6047 does not induce desensitization of the receptor when cells were treated with PN6047 (1 *µ*M) for 48 hours. In contrast, the response to SNC80 had completely desensitized at 48 hours (Supplemental Fig. 4). Desensitization was measured as inhibition of forskolin-stimulated cyclic AMP in human *δ* opioid–expressing CHO cells, and the desensitization was blocked by naltrindole.

#### Murine MIA Model of Osteoarthritis.

**Fig. 4.**
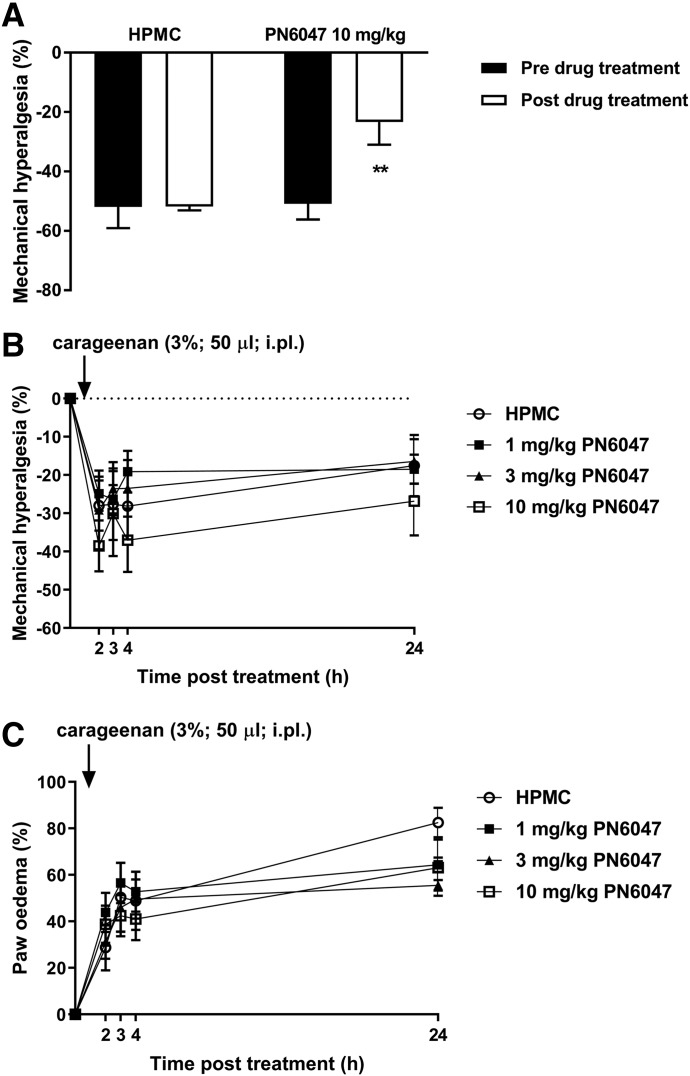
PN6047 is an effective antihyperalgesic in the mouse MIA model of osteoarthritis but not in the carrageenan model of acute inflammatory pain. (A) Mice received an intra-articular injection of MIA (25 mg/ml^−1^ in 20 *µ*l; 0.5 mg) and were allowed to recover for 7 days. Mechanonociceptive thresholds were determined prior to administration of either vehicle (HPMC, PO) or PN6047 (10 mg/kg, PO), and 60 minutes later the mechanonociceptive responses were determined (*n* = 6 to 7 mice/group). Results are expressed as means ± S.E.M., and data were analyzed with one-way ANOVA followed by Bonferroni’s modified *t* test to compare the pre- and post-treatment values (**P < 0.01). (B and C) Effect of PN6047 on carrageenan-induced acute inflammatory pain behavior and inflammation. Sixty minutes after administration of vehicle (HPMC, PO) or 1, 3, or 10 mg/kg PN6047 (PO), carrageenan (3%, 50 *μ*l) was injected into the plantar surface hind paw. Mechanonociceptive responses (B) and paw volume (C) were assessed at 2, 3, 4, and 24 hours post drug administration. Data represent the mean ± S.E.M. (*n* = 7 mice/group) and were analyzed by two-way ANOVA followed by Bonferroni’s modified *t* test to compare the pre- and post-treatment values. i.pl., intraplantar.

In the MIA model of osteoarthritis, treatment with a single dose of PN6047 reversed the MIA-induced mechanical hyperalgesia (Fig. 4A). Seven days after intra-articular injection of MIA (0.5 mg), administration of PN6047 (10 mg/kg, PO, 60 minutes) decreased the mechanical hyperalgesia (−50.9% ± 5.2% vs. −23.3% ± 7.6% hyperalgesia, pretreatment vs. post-treatment, respectively; *P* < 0.01). Vehicle (HPMC, PO, 60 minutes) alone had no effect on the extent of hyperalgesia induced by MIA (Fig. 4A).

#### Carrageenan-Induced Acute Inflammation and Inflammatory Pain Behavior.

PN6047 had no effect on either acute inflammatory pain or paw edema in the carrageenan model of acute inflammation in mice. Intraplantarly administered carrageenan (3%, 50 *μ*l, intraplantar) induced a decrease of the mechanonociceptive thresholds (Fig. 4B); this acute hyperalgesia was not affected by vehicle (HPMC, PO) or PN6047 (1, 3, 10 mg/kg, PO) administration. Similarly, carrageenan induced 30%–45% paw edema. The extent of edema was not statistically different between vehicle (HPMC, PO) or PN6047-pretreated mice (1, 3, 10 mg/kg, PO) throughout the experimental procedure (Fig. 4C).

### Behavioral Studies and Adverse Effect Assessment

#### Forced Swim Test of Depressive-Like Behavior.

**Fig. 5.**
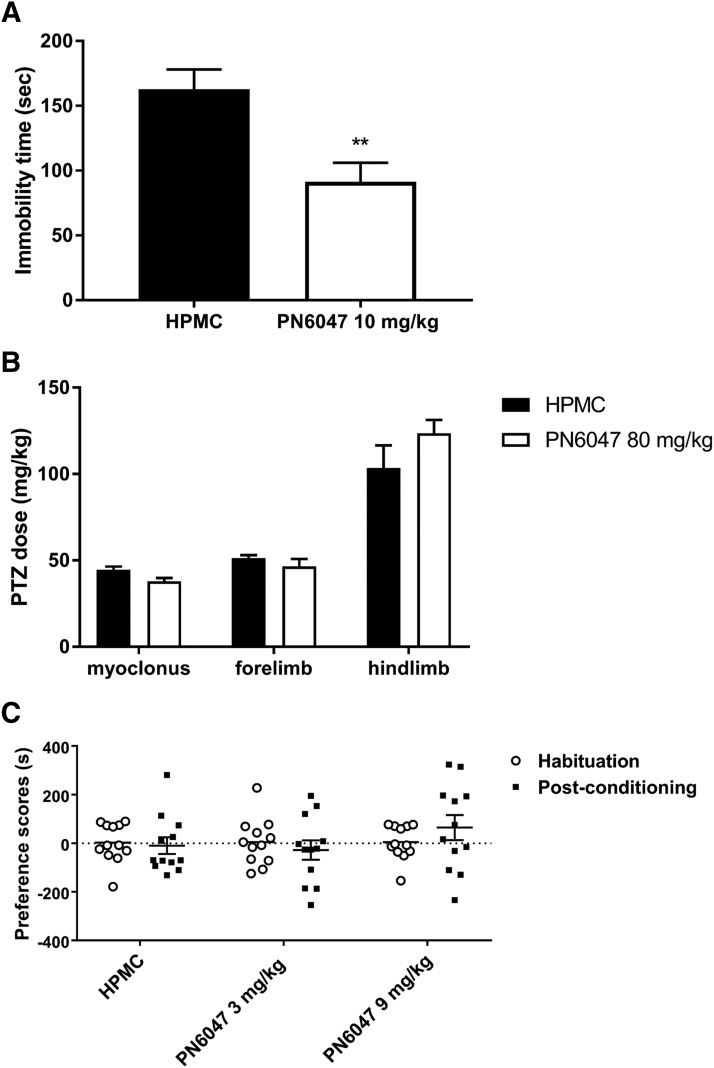
PN6047 has antidepressant activity but is not proconvulsive and does not display preference in CPP. (A) Effect of PN6047 in the mouse forced swim test of antidepressant-like activity. Immobility time in the forced swim test 60 minutes after treatment with vehicle (HPMC, PO) or PN6047 (10 mg/kg, PO). Data represent the mean ± S.E.M. (*n* = 7 to 8 mice/group). Data were analyzed with unpaired Student’s *t* test (**P < 0.01). (B) PN6047 had no effect on chemically induced seizures in the PTZ test in mice. The threshold dose of PTZ (milligrams per kilogram) required to produce myoclonic, forelimb tonic, and hindlimb tonic seizures in mice pretreated with vehicle (HPMC, PO) or PN6047 (80 mg/kg, PO) for 30 minutes (*n* = 10 to 11 mice per drug group). Data represent the mean ± S.E.M. and were analyzed by two-way ANOVA followed by Bonferroni’s post hoc test. (C) PN6047 does not induce conditioned place preference. Preference scores for rats conditioned with either vehicle (HPMC, i.p.) or PN6047 (3 or 9 mg/kg, i.p.). Data represent individual rat responses with mean ± S.E.M. overlaid (*n* = 12 rats/group) and were analyzed by Student’s *t* test for paired comparison between postconditioning vs. habituation scores.

Several lines of evidence from animal models support the utility of *δ* opioid agonists in the treatment of depression and anxiety (Pradhan et al., 2011). In the mouse forced swim test, 1 hour post drug administration, PN6047 (10 mg/kg, PO) produced a robust decrease in the time spent immobile, indicating an antidepressant-like effect (Fig. 5A; *P* < 0.01).

#### Convulsions.

Some of the first selective *δ* opioid agonists had proconvulsant properties, and PN6047 was, therefore, evaluated for proconvulsive activity. Seizure threshold was assessed by tail-vein infusion of PTZ (4 mg/min) in mice. Pretreatment with a high dose of PN6047 (80 mg/kg, PO) induced no seizure activity during the 30-minute pretreatment period, nor did it alter the subsequent dose of PTZ required to induce myoclonic (*P* = 0.866), forelimb tonic (*P* = 0.944), and hindlimb tonic seizures (*P* = 0.112) in comparison with vehicle-treated (HPMC) control mice (Fig. 5B).

#### Abuse Liability.

To determine whether PN6047 has any reward-like properties, PN6047 was tested in the CPP model (Fig. 5C). Rats conditioned with PN6047 (3 or 9 mg/kg) showed no change in preference compared with habituation (Fig. 5C; 3 mg/kg PN6047, i.p., *P* = 0.56; 9 mg/kg PN6047, i.p., *P* = 0.24). In contrast, rats conditioned with heroin (1 mg/kg, s.c.) showed a significant increase in preference for the drug-paired compartment (Supplemental Fig. 5A; *P* < 0.05). Of note, locomotor activity was also assessed in these experiments, and no significant difference was observed between any of the experimental groups (Supplemental Fig. 5B).

#### Acute Nociception and Respiratory Depression.

**Fig. 6.**
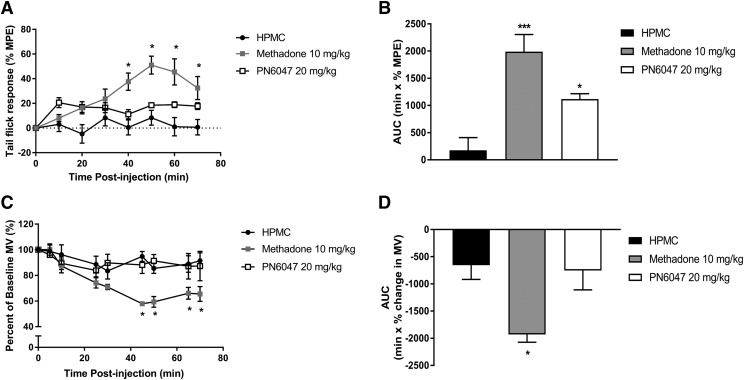
PN6047 has limited acute analgesic activity but does not depress respiration. (A and B) PN6047 at a high dose had marginal efficacy to affect tail withdrawal latencies in mice. (C and D) This high dose of PN6047 did not induce respiratory depression as measured by percentage change in minute volume (MV). In (B) and (D), the data in (A) and (C) have been recalculated and plotted as the area under the curve (AUC). The AUC for the percentage change in minute volume has been calculated for each individual animal before the mean AUC has been calculated. Mice were administered vehicle (HPMC, PO), PN6047 (20 mg/kg, PO), or methadone (10 mg/kg, PO) 60 minutes prior to either thermal nociceptive testing or whole-body plethysmography (*n* = 5 mice/group). Methadone significantly affected both tail withdrawal latencies and respiratory depression. Data represent the mean ± S.E.M. and were analyzed using two-way ANOVA followed by Bonferroni’s post hoc test in (A) and (C) and a one-way ANOVA with Bonferroni’s post hoc test in (B) and (D) (*P < 0.05). MPE, maximal possible effect.

For the *δ* opioid receptor, there is limited evidence to suggest that agonists are effective in acute nociception and have the potential to induce respiratory depression, both of which are typically thought to be mediated via activation of the *µ* opioid receptor. To evaluate the effect of PN6047, an additional group of mice treated with the *µ*-selective agonist methadone were included for direct comparison. Assessment of acute antinociceptive activity of PN6047 (20 mg/kg, PO) using the tail-flick assay revealed that PN6047 has limited efficacy in acute thermal nociception (Fig. 6, A and B). While the effect of PN6047 was not significant in comparison with vehicle-treated mice at specific time points, a composite of the data, calculated from the area under the curve, shows a small but significant effect of PN6047 (*P* < 0.05). In comparison, methadone (10 mg/kg, PO) substantially reduced tail withdrawal latencies (*P* < 0.001).

PN6047 (20 mg/kg, PO) had no effect on respiration in mice. Respiratory parameters were monitored using whole-body plethysmography for 70 minutes post drug administration. Methadone significantly depressed respiration (*P* < 0.01), whereas in PN6047-treated mice, the minute volume did not differ from vehicle-treated (HPMC) mice at any of the time points investigated or when the area under the curve was calculated (Fig. 6, C and D).

## Discussion

Increasing evidence implicates the *δ* opioid receptor as an attractive therapeutic target for various chronic pain states. Here, we report on PN6047, a novel small-molecule *δ* opioid receptor agonist. The data presented demonstrate that PN6047 is an orally bioavailable, G protein–biased, and *δ*-selective agonist with potent antihyperalgesic efficacy in preclinical models of chronic pain. Notably, PN6047 does not appear to have proconvulsive activity or induce analgesic tolerance.

PN6047 shows a differential signaling profile compared with the prototypical agonist SNC80 in vitro. PN6047 elicited a maximal response in the BRET G protein activation assay equivalent to that of SNC80 but with greater potency (10-fold difference). Given that SNC80 and PN6047 were determined to have similar affinities for the *δ* opioid receptor in binding studies, our results indicate that PN6047 is a more efficacious ligand with respect to G protein activation. In contrast to the response in the G protein assay, PN6047 is a partial agonist for both arrestin-2 and arrestin-3 recruitment, being a particularly weak recruiter of arrestin-2. Examining bias across the different signaling pathways revealed that PN6047 is significantly biased toward G protein activation over arrestin recruitment, relative to SNC80. SNC80 was selected as our reference ligand, as it is often regarded as a prototypical ligand and it has been well characterized in many in vitro and behavioral assays (Jutkiewicz et al., 2005; Pradhan et al., 2010; Dripps et al., 2018). The peptide agonist DADLE was also shown to be G protein–biased in our studies. Interestingly, another synthetic opioid peptide, DPDPE ([D-Pen^2^,D-Pen^5^]enkephalin), has recently been reported as being biased toward G protein activation relative to arrestin recruitment when SNC80 is used as a reference compound (Stanczyk et al., 2019). ARM390 has previously been reported as a low-internalizing *δ* opioid agonist, and this has been suggested to arise as a consequence of limited arrestin recruitment (Pradhan et al., 2016). While ARM390 was a deemed to be a full agonist in our assays, it did exhibit lower potency than the other agonists and was significantly less potent than SNC80 in the arrestin recruitment assays. Although the maximum response to ARM390 in the arrestin recruitment assays was not statistically different from that of SNC80, this is likely to be a result of the curve-fitting process on individual experiments. In line with its G protein efficacy, PN6047 elicited ERK activation of magnitude and duration equivalent to that seen with SNC80. Similarly, in relation to its limited ability to recruit arrestins, PN6047 was a partial agonist with respect to internalization.

The antihyperalgesic properties of PN6047 in the SNL and MIA model are similar to those of several other nonpeptide *δ* opioid receptor agonists, such as SNC80, ARM390, and ADL5859, which have been shown to be effective in both neuropathic pain models and chronic inflammatory pain models (Bilsky et al., 1995; Codd et al., 2009; Pradhan et al., 2009). Although PN6047 did not completely reverse the mechanical hyperalgesia in either the SNL model or the MIA model, this is potentially due to the use of a submaximal dose of PN6047 in both experiments. Higher concentrations of PN6047 in different chronic pain models will need to be evaluated in the future. The antihyperalgesic action of PN6047 in the SNL model was inhibited by administration of either the selective *δ* antagonist naltrindole or the peripherally restricted antagonist naloxone methiodide, indicating that there is a peripheral component to the antihyperalgesic effect of PN6047. These findings agree with previously published data that have shown peripheral *δ* opioid receptors are expressed by both unmyelinated and myelinated sensory nerves (Bardoni et al., 2014) and are important for mediating the analgesic effects of *δ* opioid agonists (Scherrer et al., 2009; Stein et al., 2009; Gaveriaux-Ruff et al., 2011).

Repeated administration of PN6047 did not induce analgesic tolerance. This is an important finding, as the onset of analgesic tolerance has been a major drawback in the development of *δ* opioid agonists. This lack of tolerance to PN6047 contrasts with the effects of repeated, once-daily administration of SNC80, ARM390, and KNT-127, all of which induce analgesic tolerance following prolonged dosing over days, although this is thought to occur via different molecular mechanisms (Jutkiewicz et al., 2005; Pradhan et al., 2009, 2016; Nozaki et al., 2014). We observed no change in the analgesic efficacy of PN6047 over a 16-day dosing regimen. For comparison, a recent study reported that repeated treatment of mice (once daily; 10 mg/kg) with SNC80 resulted in analgesic tolerance within 3 days (Vicente-Sanchez et al., 2018). The lack of tolerance to PN6047 may in part arise as a consequence of its limited ability to induce internalization. For the *δ* opioid receptor, there is now a well established link between receptor trafficking fates and the onset and duration of tolerance (Pradhan et al., 2009, 2010; Vicente-Sanchez et al., 2018). Furthermore, in arrestin-2 knockout animals, analgesic tolerance to SNC80 is attenuated (Vicente-Sanchez et al., 2018).

Despite having significant analgesic action in rodent models of chronic pain, administration of PN6047 had no effect on either acute inflammatory pain or paw edema following intraplantar injection of carrageenan. This indicates that the action of PN6047 is selective for chronic pain states, and that the drug has minimal therapeutic potential for acute inflammatory pain states. As with some of the preclinical pain models, the efficacy of different *δ* opioid agonists in the carrageenan-induced inflammatory pain model appears to be ligand-dependent. For example, SNC80 has been shown to reverse carrageenan-induced tactile allodynia but not carrageenan-induced thermal hyperalgesia in rats (Kouchek et al., 2013), whereas another small-molecule *δ* opioid agonist, SB-235863, potently reversed thermal hyperalgesia in rats resulting from a carrageenan-induced inflammatory response (Petrillo et al., 2003).

In the forced swim test, PN6047 decreased immobility, consistent with the well established antidepressant-like effects of *δ* receptor agonists (Pradhan et al., 2011). Various studies to date have documented the potential of *δ* opioid agonists as antidepressant drugs. For example, SNC80, ADL5859, and KNT-127 all inhibit depressive-like behavior, and these effects were reported to be comparable to that of prototypical antidepressant drugs, including selective serotonin reuptake inhibitors and tricyclic antidepressants (Saitoh et al., 2004, 2011; Le Bourdonnec et al., 2008). On the other hand, in a limited clinical trial with the *δ* opioid agonist AZD2327 in anxious, depressed patients, although the drug’s effects overall failed to reach significance, it was concluded that AZD2327 had larger potential anxiolytic than antidepressant activity (Richards et al., 2016).

The observation that some of the earlier developed *δ* opioid agonists can cause convulsions has limited their clinical development (Comer et al., 1993; Broom et al., 2002). This effect has been demonstrated to be mediated via activation of the *δ* opioid receptor through the use of *δ* opioid antagonist and *δ* receptor knockout mice (Nagase et al., 1998). However, other ligands have subsequently been developed that are devoid of convulsive activity, including ADL5859, JNJ-20788560, ARM390, and KNT-127 (Le Bourdonnec et al., 2008; Codd et al., 2009; Saitoh et al., 2011; Chung et al., 2015). In line with these agonists, we observed no evidence of proconvulsive activity of PN6047 at a dose 80-fold greater than that required for its antihyperalgesic activity. These data provide further support for the notion that the convulsive activity of certain *δ* opioid agonists is ligand-specific and not a property common to this drug class. The signaling mechanisms that mediate the proconvulsive activity of some *δ* agonists are not yet fully understood. While some of the aforementioned agonists that are devoid of proconvulsive activity are also reported to be low-internalizing agonists and are not thought to engage arrestin efficiently, SNC80-induced convulsions were actually potentiated in arrestin-2 knockout mice (Dripps et al., 2018; Vicente-Sanchez et al., 2018), suggesting that further work is required to determine the contribution, or lack of contribution, of arrestin in mediating seizure behavior.

Assessment of other opioid-related adverse effects also revealed a beneficial behavioral profile of PN6047. PN6047-treated rodents did not exhibit typical *µ* opioid receptor–related behaviors, including respiratory depression or conditioned place preference. In fact, there is little evidence for any rewarding properties of *δ* opioid agonists in rodents (van Rijn et al., 2012) or nonhuman primates (Hudzik et al., 2014).

PN6047 had a small but significant effect on acute nociception (tail flick) but only when comparisons were made on the composite data. Concurrent experiments with either methadone or heroin demonstrated that these agonists exhibited characteristic *µ* opioid–mediated behaviors. In agreement with these finding, the *δ* opioid agonist JNJ-20788560 has been shown not to induce respiratory depression or precipitate withdrawal behaviors consequent to opioid antagonist administration, which is indicative of adverse effects and physical dependence (Codd et al., 2009). Thus, PN6047 does not induce respiratory depression or possess abuse liability, two of the major adverse effects currently associated with *µ* opioid agonists. These finding are supported by the cellular studies assessing opioid receptor selectivity whereby PN6047 did not elicit a response in cells expressing either the *µ* opioid receptor or *κ* opioid receptor, and PN6047 did not inhibit the response of prototypical agonists at either of these receptors.

The in vivo profile of PN6047 shown here demonstrates that *δ* opioid ligands that are potent antihyperalgesics yet devoid of troublesome on-target side effects represent a feasible approach for the treatment of chronic pain states and depression. Importantly, PN6047 is a G protein–biased ligand that is a full agonist for G protein activation. The in vivo significance of a G protein–biased *δ* agonist remains to be fully explored. Although we have provided evidence of a ligand with an improved therapeutic profile, it cannot be determined at present if this arises as a direct consequence of the biased signaling or perhaps due to the limited arrestin recruitment, particularly with respect to arrestin-2, which has been shown to play a role in some of the unwanted behavioral effects observed for other *δ* opioid agonists (Pradhan et al., 2016; Dripps et al., 2018; Vicente-Sanchez et al., 2018). Certainly, there is evidence in the literature to suggest that G protein–biased *δ* opioid ligands or *δ* opioid ligands that weakly engage arrestin would offer clinical superiority (Pradhan et al., 2009, 2010; Vicente-Sanchez et al., 2018). A greater understanding of the signaling pathways that underlie the different behavioral responses to *δ* opioid agonists together with knowledge of the structural basis for this differential signaling will provide further insight into the potential utility of this drug class for treating chronic pain and emotional disorders. Collectively, our results provide further support for the development of G protein–biased *δ* agonists as a strategy for improving the therapeutic profile of *δ* opioid agonists.

## Abbreviations

BRET: bioluminescence resonance energy transfer
CPP: conditioned place preference
DADLE: [D-Ala^2^, D-Leu^5^]-enkephalin
DAMGO: [D-Ala2, N-MePhe4, Gly-ol]-enkephalin
DMEM: Dulbecco’s modified Eagle’s medium
DOPr: *δ* opioid receptor
GFP: Green Fluorescent Protein
HA: hemagglutinin
HEK293: human embryonic kidney 293
HPMC: hydroxypropyl methylcellulose
MIA: mono-iodoacetate
pERK: phosphorylated ERK
PN6047: 3-[[4-(dimethylcarbamoyl)phenyl]-[1-(thiazol-5-ylmethyl)-4-piperidylidene]methyl]benzamide
PO: by mouth
PTZ: pentylenetetrazol
Rluc: Renilla Luciferase
SNL: sciatic nerve ligation
